# Tumor LDH-A expression and serum LDH status are two metabolic predictors for triple negative breast cancer brain metastasis

**DOI:** 10.1038/s41598-017-06378-7

**Published:** 2017-07-20

**Authors:** Tieying Dong, Zhaoliang Liu, Qijia Xuan, Zhuozhong Wang, Wenjie Ma, Qingyuan Zhang

**Affiliations:** 1 Department of Internal Medicine, The Third Affiliated Hospital of Harbin Medical University, Haping Road 150 of Nangang District, Harbin, Heilongjiang Province 150081 China; 2 Cancer Research Institute, Harbin Medical University, Harbin, China; 3 Cancer Research Institute of Heilongjiang, Harbin, China; 4 Department of Epidemiology and Biostatistics, Harbin Medical University, Harbin, China

## Abstract

There are limited therapeutic methods for triple negative breast cancer in the clinic, which is easy to progress into the brain to form metastatic lesions and evolve into the terminal stage. Because both the primary cancer and the brain metastasis have high glycolysis, we hypothesize that lactate dehydrogenase (LDH), which catalyzes the final step of glycolysis, may be a predictor, as well as a treatment target, for breast cancer brain metastasis. Therefore, the expression of LDH-A was detected on 119 triple negative breast cancer tissues with immunohistochemistry, and the serum LDH levels were also measured. Our results showed that the LDH-A expression inside the tumor was significantly higher than the matched normal tissues. Tumor LDH-A expression, serum LDH status, and the slope of serum LDH status were closely associated with triple negative breast cancer brain metastasis and brain metastasis free survival. This study indicates that tumor LDH and serum LDH status are two predictors for triple negative breast cancer brain metastasis.

## Introduction

Breast cancer has the highest cancer incidence in women and is one of the leading causes of mortality globally^1^. Triple negative breast cancer (TNBC) is characterized with the negative expression of estrogen receptor, progesterone receptor, and human epidermal growth factor receptor 2. There are limited effective therapeutic methods for TNBC patients except for chemotherapy due to the lack of targets^2^. TNBC is often associated with more aggressive behaviors and a higher mortality than the other subtypes of breast cancer due to the recurrence and metastasis, the main reasons for its incurable nature^3–7^. About 10–30% of patients with metastatic breast cancer develop brain metastasis^8^. The incidence of breast cancer brain metastasis (BCBM) in TNBC patients is about 20%^9, 10^. Traditional treatment options have minimal efficacy for BCBM, and the overall survival is short with a median survival time of approximate 5 months despite the use of whole-brain radiotherapy. However, the incidence of BCBM is increasing with the improved systemic management of the disease and the prolongation of survival^8, 11–13^. Therefore, it is imperative to gain a better understanding of the nature and functionality of breast cancer cells that cause brain metastasis to develop effective regimens^14^.

Recently, the altered energy metabolism has been realized to be one of the hallmarks of cancer^15^ and is linked to cancer metastasis, drug resistance, and patient survival. Targeting cellular metabolism is becoming a promising strategy to make therapy effective and prolong the patient survival in cancer treatment^2^. TNBC mostly exhibit a higher level of glycolysis, which needs higher expression levels of related enzymes^7, 16^. Increased activity of enzymes involved in glycolysis, like pyruvate kinase 2, glucose-6-phosphate dehydrogenase, and lactate dehydrogenase A (LDH-A), has been studied, and their expression may affect cancer cell growth^5, 17^. A lot of articles had reported breast cancer brain metastatic cells had increased expression of enzymes associated with glycolysis and oxidative phosphorylation pathways, indicating that the brain metastatic cells derive energy from glucose^7, 14, 16, 18^ and may have the higher expression of glycolysis-related genes than other metastasis sites. The glycolytic level in brain metastasis is not only higher than other metastatic sites, such as lung, liver, and bone, but also higher than the primary cancer. The high activity of glycolysis in brain metastatic cells attracts our attention to further explore its potential in the cancer prognosis and therapeutics.

Lactate dehydrogenase (LDH) is a tetrameric enzyme comprising two major subunits A and/or B, resulting in five isozymes (A4, A3B1, A2B2, A1B3, and B4) that can catalyze the forward and backward conversion of pyruvate to lactate. The conversion of pyruvate to lactate, catalyzed by LDH-A, is the final step of aerobic glycolysis. LDH-A is a vital metabolic enzyme that is associated with cancer development, invasion, and metastasis^19–22^. LDH-A has been reported to correlate with clinicopathologic characteristics and survival outcome of multiple cancers^23–26^. The inhibition of LDH-A has an anti-proliferative effect on primary breast tumors^27^. However, there has been little studies investigating the effect of LDH-A on the brain metastasis of breast cancer or evaluating noninvasive methods monitoring LDH-A. The dynamic metabolic changes can be reflected by an elevated serum LDH level^28^. Elevated LDH has been recognized as a poor prognostic indicator in cancer for many years^29–32^, and it also has been incorporated in prognostic scores for several types of cancer^33^. However, the prognostic impact of LDH on breast cancer brain metastases is unclear^34–36^. Based on the above research, we hypothesize that LDH would be a predictor for BCBM.

## Result

### Patients and clinical characteristics

The information of 119 patients was obtained from The Third Affiliated Hospital of Harbin Medical University. The median age of these female patients was 48 (ranging from 30 to 68), the survival duration from the surgical operation to the development of brain metastasis ranged from 11 to 117 months with a median of 71 months. The patients without brain metastasis were censored at the time of the last follow-up. Clinicopathological characteristics and their relationships to LDH/LDH-A, brain metastasis, and survival are shown in Table 1. Tumor sizes and Ki-67 positive percentages are significantly different (p = 0.023; p = 0.019) between the LDH-A positive cancer group and negative group (Table 1).

**Table 1:** The associations between clinicopathological characteristics and LDH/LDH-A status. ^b^by Kaplan-Meier analysis ^a^by two-sided Pearson’s exact test *P refers to positive,*N refers to negative.

Characteristics		Tissue LDHA expression		p value^a^	Baseline Serum LDH –		p value^a^	Method A				p value^a^	Method B		p value^a^	Brain metastases		p value^a^		Survival p value^b^
		N*	P*		Normal	Elevated		Persis-tently normal	Impr-oved	Deteri-orated	Persistently elevated		Low	High		N*	P*			
Age	≤48 (median age)	19	41	0.236	48	12	0.785	20	5	28	7	0.551	34	26	0.388	20	40	0.166		0.259
	>48	13	46		46	13		24	3	22	10		38	21		27	32			
Menopause status	Pre-	18	45	0.661	49	14	0.73	22	5	27	9	0.765	38	25	0.965	20	43	0.067		0.192
	Post-	14	42		45	11		22	3	23	8		34	22		27	29			
BMI	<18.5	2	7	0.692	6	3	0.301	2	2	4	1	0.612	6	3	0.923	3	6	0.827		0.806
	18.5–24.9	19	44		53	10		23	2	30	8		38	25		24	39			
	>24.9	11	36		35	12		19	4	16	8		28	19		20	27			
Tumor size (cm)	≤2	6	4	0.023	10	0	0.118	4	0	6	0	0.16	7	3	0.521	5	5	0.514		0.22
	>2	26	83		84	25		40	8	44	17		65	44		42	67			
Histology grade	II	19	60	0.326	65	14	0.216	33	6	32	8	0.195	48	31	0.936	31	48	0.936		0.397
	III	13	27		29	11		11	2	18	9		24	16		16	24			
Nodal status	N0	18	42	0.44	50	10	0.241	18	3	32	7	0.091	36	24	0.323	23	37	0.794		0.799
	N+	14	45		44	15		26	5	18	10		36	23		24	35			
Ki67	<14%	21	36	0.019	46	11	0.661	22	5	22	6	0.415	34	23	0.855	26	31	0.191		0.108
	≥14%	11	51		48	14		20	3	28	11		38	24		21	41			
P53	N*	24	61	0.601	66	19	0.569	30	6	36	13	0.918	49	36	0.313	34	51	0.859		0.87
	P*	8	26		28	6		14	2	14	4		23	11		13	21			
Brain metastases	N*	18	29	0.023	40	7	0.186	29	3	11	4	<0.001	32	15	0.172					
	P*	14	58		54	18		15	5	39	13		40	32						

### Tumor LDH-A expression is higher than the matched normal tissues

**Figure 1:**
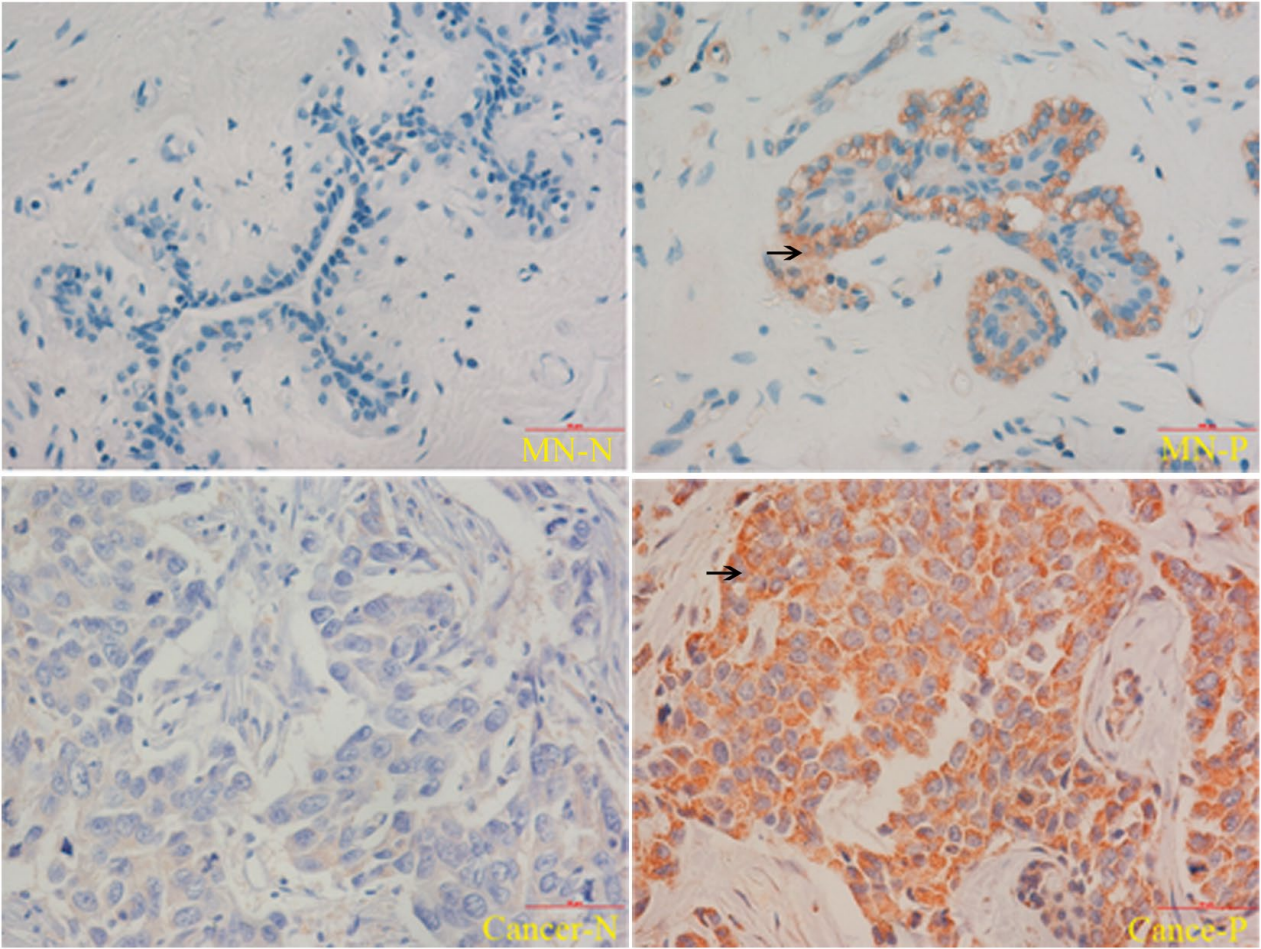
IHC of LDH-A in the breast cancer tissues and matched normal tissues. Patients showed the markedly different expression of LDH-A in the positive and negative cancer samples. MN-N: the matched normal tissue with LDH-A negative expression; MN-P: the matched normal tissue with LDH-A positive expression; Cancer-N: the cancer tissue with LDH-A negative expression; Cancer-P: the cancer tissue with LDH-A positive expression. The black arrows show LDH-A positive cells.

We obtained the tissue samples from the 119 enrolled patients, which included both the breast cancer tissues and the matched normal tissues. The tissue samples were stained under the same condition (Fig. 1). The paired sample *t*-test was applied to determine the significant difference (p < 0.001) between the two groups. A great proportion of cases (up to 57%, 68/119) showed negative LDH-A expression in the matched normal tissue and positive expression in the cancer tissue, while only 6% (7/119) of the whole samples showed positivity in the matched normal tissues and negativity in the cancer tissues.

### Tumor LDH-A expression levels do not agree with the baseline serum LDH levels

We detected the expression of LDH-A in breast cancer tissues and the serum LDH levels and used the two sets of data to analyze the agreement between them. The Kappa test and McNemar test gave the result of Kappa = 0.125, p < 0.001.

### Tumor LDH-A expression, as well as serum LDH status categorized by Method A, is associated with brain metastasis status

Method A and Method B are two ways of determining serum LDH status. The details of the methods are described in the section of “Materials and Methods”. The associations of brain metastatic status with tumor LDH-A expression, pre-operational serum LDH level, and serum LDH status (including the status evaluated by Method A and Method B) were calculated by chi-square test. Tumor LDH-A expression and serum LDH status categorized by Method A were found to be significantly associated with brain metastasis (p = 0.023, p < 0.001, respectively), but not the baseline serum LDH levels nor the LDH status determined by Method B (Table 1).

### Tumor LDH-A expression, as well as the serum LDH status determined by both Method A and Method B, is associated with the brain metastasis free survival

**Figure 2:**
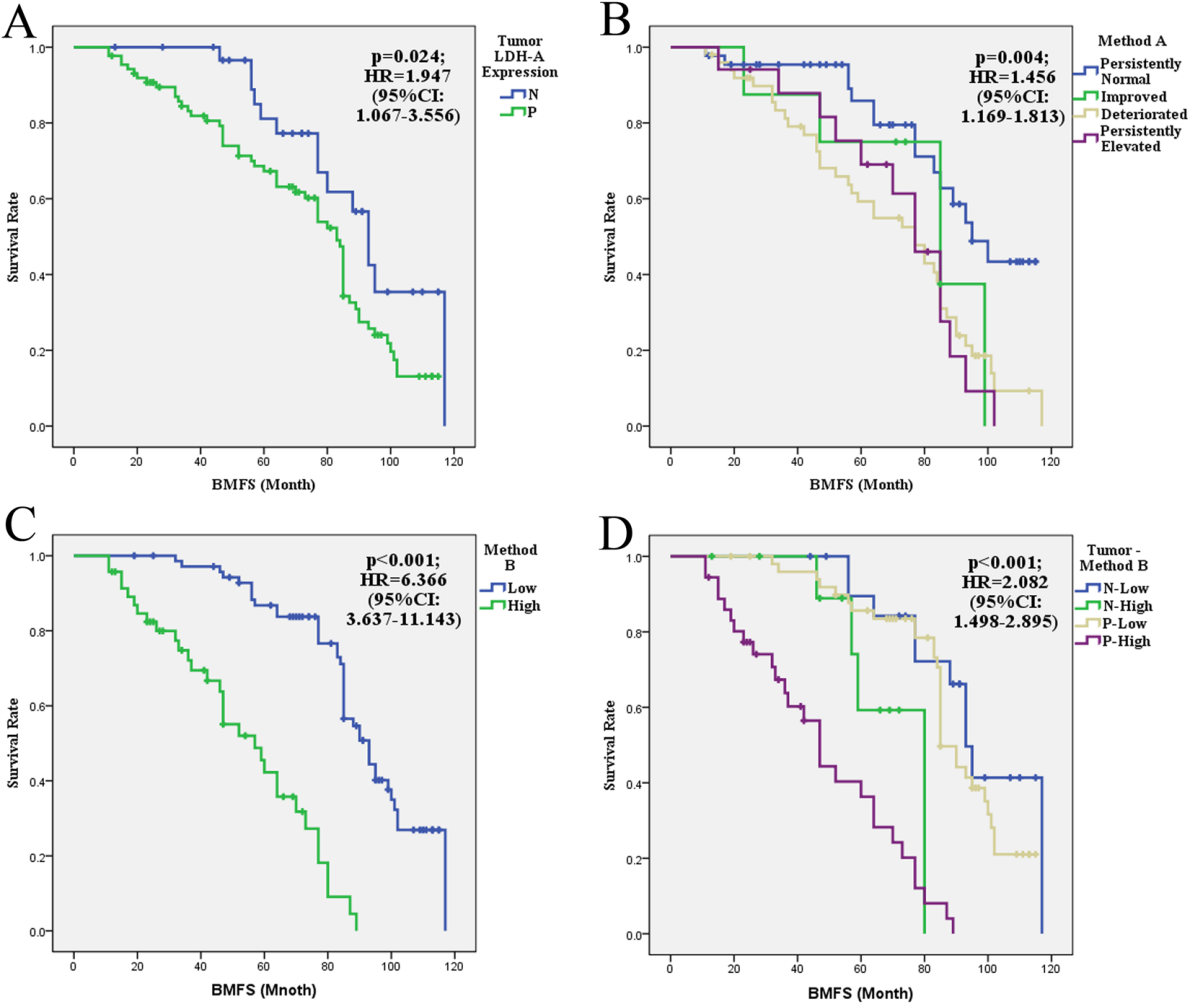
Kaplan–Meier curves for BMFS according to tumor LDH-A expression and serum LDH. (**A**) The Kaplan–Meier curves for BMFS according to tumor LDH-A expression; (**B**) according to serum LDH status determined by Method A; (**C**) according to serum LDH status determined by Method B and (**D**) according to the combination of tumor LDH-A expression and serum LDH status determined by Method B (P refers to the patients with LDH-A positive expression, N refers to the patients with LDH-A negative expression).

Brain metastasis free survival (BMFS) refers to the period from the diagnosis to the development of brain metastasis or the last follow-up. Kaplan-Meier method was employed to analyze the associations of BMFS with tumor LDH-A expression, baseline serum LDH levels, and the serum LDH status determined by Method A and Method B. The Cox’s proportional hazards regression model was used to evaluate the hazard rate (HR). Tumor LDH-A expression, as well as the serum LDH status determined by Method A and Method B, was significantly associated with BMFS (p = 0.024, p = 0.004, p = 0.001, respectively) (Fig. 2). The HR of positive tumor LDH-A expression combined with high serum LDH (determined by Method B, ≥5.0 U/L/month) was up to 6.454 (95%CI:3.518–11.838; p < 0.001) compared with positive tumor LDH-A expression combined with low serum LDH. However, we didn’t observe a significant association when we used the negative tumor LDH-A expression combined with the two groups of Method B. There was only two individuals in the subgroup of negative tumor LDH-A expression and deteriorated LDH status when the patients were categorized with Method A, so we did not further analyze the data by combining the tumor LDH-A expression and Method A LDH status.

## Discussion

Brain metastasis is the worst complication of breast cancer due to the short survival of the patients and limited therapeutic regimens. High glycolysis activity is a prominent feature of brain metastasis accompanied by high expression levels of glycolysis-related proteins. Patients with brain metastasis consist of more TNBC cases as shown in previous reports^16, 18, 37^. Therefore, all the patients in this study were with the molecular phenotype of triple negative breast cancer and with the histopathology of infiltrating ductal carcinoma, had accepted modified radical mastectomy, and had no diabetes. LDH-A expression depends on the functions of the tissues and the increases in responses to tissue injury, necrosis, hypoxia, and so on. The tissues in this study had not been injured before modified radical mastectomy. The elevated serum LDH is not only prevalent in human malignancies, but also related to tissue injury, hemolysis, and hepatic failure. Peripheral blood samples with relative normal transaminase levels and without hemolysis were collected^38–40^.

As an important checkpoint enzyme catalyzing the final step of glycolysis, LDH-A upregulation not only facilitates the anaerobic glycolysis in tumor cells and reduces their dependency on oxygen, but also produces more lactic acid. The upregulated glycolysis is an evolution result of cancer cells for the adaptation to hypoxia. However, it also has significant negative effects on normal cells for the increased lactic acid production leads to the significantly decreased extracellular pH. Normal cells, exposed in this low extracellular pH microenvironment for a long time, will enter necrosis or apoptosis through caspase-3-dependent and p53-dependent mechanisms^41, 42^. On the other hand, lactic acid accumulation induces the degradation of the extracellular matrix, destroys the adjacent normal cell populations, and promotes angiogenesis. Therefore, the lactic acid accumulation catalyzed by LDH-A helps cancer cells break down the barriers, which are comprised of normal cells and extracellular matrix and are protective mechanisms against cancer metastasis, to fulfill the first step of cancer cell migration.

The LDH-A expression inside the cancer is a reflection of metabolic rates, and a high metabolic rate is the basic requirement of tumor proliferation. We found the LDH-A expression was positively correlated to tumor sizes, which indicated that it might influence tumor proliferation. Meanwhile, Ki-67 showed a distinctive association with tumor LDH-A expression. The proportion of Ki67 positive cancer cells was significantly reduced in the LDH-A negative tumor samples. This observation was consistent with previous studies, which showed that the small number of Ki67-positive cells are related to the slow growth of LDH-A deficient tumors. This phenomenon was also observed in the LDH-A knockdown tumor tissue in an *in vitro* study^37^.

The distinctively high expression of LDH-A in the breast cancer tissues compared with the matched normal tissues might indicate that the expression of LDH-A is a indicator of the malignancy degree. Another immunohistochemical study revealed that LDH-A is primarily expressed in cancer cells, whereas normal and carcinomatous tissues have similar levels of LDH-B, which is a member of LDH family^43^. LDH-B predominates in the tissues with an aerobic metabolism like the heart, while LDH-A is mainly present in the tissues with considerable anaerobic metabolism, such as the skeletal muscle, liver, and tumor. Furthermore, in a small patient cohort, the LDH-B levels were consistently low compared to the LDH-A levels^44^. These pieces of evidence enable LDH-A to be used as a biomarker for many malignancies^45–47^.

Tumor LDH-A expression, as a predictive biomarker, is the most straightforward. However, it will take a long time from the determination of tumor LDH-A expression to the development of brain metastasis. Serum LDH status can monitor the disease progression at the real time. Taken together, both LDH determination methods have their advantages and disadvantages. LDH-A expression inside the cancer tissue is not consistent with the baseline serum LDH levels in this study, which may indicate tumor LDH-A expression and serum LDH levels are two separated predictors. This result coincides with the view of Koukourakis *et al*. that the serum LDH-A levels were not correlated with cancer tissue levels in 71% of LDH-A positive cases^48^.

Serum LDH, which monitors the disease progression in the whole period, also showed significant associations with breast cancer brain metastasis. Serum LDH status categorized by Method A shows positive results, while the baseline serum LDH level has not. This result might be due to the fluctuation of serum LDH levels compared with the tumor LDH-A. Along with the enlarging tumor volume, glycolysis enhances, and the consequent increase in LDH expression leads to the elevation of serum LDH levels. On the other hand, the LDH released from the necrotic cancer tissue elevates the serum LDH level when the tumor volume exceeds the capacity of blood supply. Therefore, the tumor LDH-A levels and serum LDH level are two independent predictors for BCBM.

Measuring serum LDH levels is a safe and simple detection method compared with using biopsy. We show two methods of evaluating serum LDH status: Method A takes all serum LDH measurements during the disease progression, and Method B quantifies the serum LDH status at specified detection time points. Method A is better than Method B in predicting the occurrence of brain metastasis since the serum LDH levels evaluated by Method A shows relevance with brain metastasis but not Method B. The combination of tumor LDH-A expression and serum LDH levels evaluated by Method B shows a better prediction for BMFS in the LDH-A positive group, which suggest that Method B should be used when tumor LDH-A expression is positive. In the analyses of the relationship between tumor LDH-A expression or serum LDH levels with brain metastasis, both of them show a distinct association, which tells us both the indices could be predictors of breast cancer brain metastasis.

In summary, our result supports that LDH-A should be inhibited for BCBM prevention and treatment. The distinctively high expression of LDH-A in the breast cancer tissues is a indicator of the malignancy degree. As tumor LDH-A expression and serum LDH levels are two separated predictors, Method A is better than Method B in predicting the occurrence of brain metastasis, but the combination of tumor LDH-A expression and Method B shows a better prediction for BMFS in the LDH-A positive patients. We proved that tumor LDH-A expression, as well as serum LDH status, is associated with brain metastasis status.

## Materials and Methods

### Patients and tissue samples

119 formalin-fixed paraffin-embedded breast cancer sections were obtained from The Third Affiliated Hospital of Harbin Medical University. These triple-negative patients were diagnosed between March 2005 and March 2015, who received standard treatment including chemotherapy and/or radiotherapy and had complete medical records. Furthermore, the patients were firstly diagnosed as triple-negative infiltrating ductal breast cancer without any severe systemic diseases or combined tumors and did not receive any treatments before the surgical operation. This study was approved by the Ethics Committee of the Third Affiliated Hospital of Harbin Medical University. Informed consent was obtained from all patients. All procedures were performed in accordance with the rules and guidelines of the Tumor Research Institute of Heilongjiang, Harbin, China. All participants in this study had signed informed consents.

### Immunohistochemistry (IHC)

Formalin-fixed paraffin-embedded breast cancer sections were cut into four micrometer-thick sections and mounted on a slide for immunohistochemical staining. They were dewaxed, incubated in saline sodium citrate (pH = 7.0) for 1 min in pressure heating environment for antigen retrieval, then soaked in 3% H_2_O_2_ solution for 30 min. Mouse monoclonal LDHA antibody (1:200, OriGene, USA) were applied at 4 °C overnight in humid chambers, followed by the secondary antibody at room temperature for 25 min. Then the color was developed by DAB. The stained specimens were reviewed by two pathologists independently. At least five visual fields were observed for each section under high power lens (×400) to calculate the percentage of positive cells (from an undetectable level (0%) to a homogeneous staining (100%)), and the intensity of staining was scored (1, weak staining; 2, moderate staining; and 3, strong staining). The scores were further calculated by multiplying the percentage of positive cells by the intensity (ranged from 0 to 300). The final score ≤100 was considered as the negative expression, and the score >100 was considered as the positive expression^49^.

### Serum LDH measurement

Blood samples from a peripheral vein puncture were collected before the operation and at each visit during the chemotherapy and follow-up period until brain metastasis development or the last follow-up. Hitachi Modular 7600 Chemistry Analyzer measures the LDH level of blood sample by spectrophotometry. LDH catalyzes lactates to pyruvates. During the reaction, NAD+ is reduced to NADH. NADH has an absorption peak at 340 nm wavelength, so the reaction increases the absorbance at 340 nm. The concentration of LDH can be determined according to the changes in absorbance at 340 nm. In the experiment, we first collected the blood samples from a peripheral vein puncture into vacutainer tubes (BD Vacutainer® SST^TM^, 367983). Then, the blood samples were centrifuged at 3000 r/min for 5 min (Hitachi CT15RE Centrifuge) and put into the Hitachi Modular 7600 Chemistry Analyzer. The analyzer measured the LDH levels automatically. Based on the serum LDH levels before the treatment, a value of >246 U/L was considered as elevated level.

### The evaluation of serum LDH status

Serum LDH levels were measured at every visit after the modified radical mastectomy until the development of brain metastasis or the last follow-up. The alteration status of serum LDH was evaluated in two ways. In the first method (Method A), the patients was classified into four subgroups according to their serum LDH levels: persistently normal, improved (the patient’s LDH levels decreased from an elevated level to the normal level), deteriorated (the patient’s LDH levels increased from the normal level to an elevated level), and persistently elevated. The baseline was the LDH level measured before the operation, and the LDH level was termed “elevated” at the terminal point if more than half of the measurements were higher than the baseline during the chemotherapy and follow-up.

**Figure 3:**
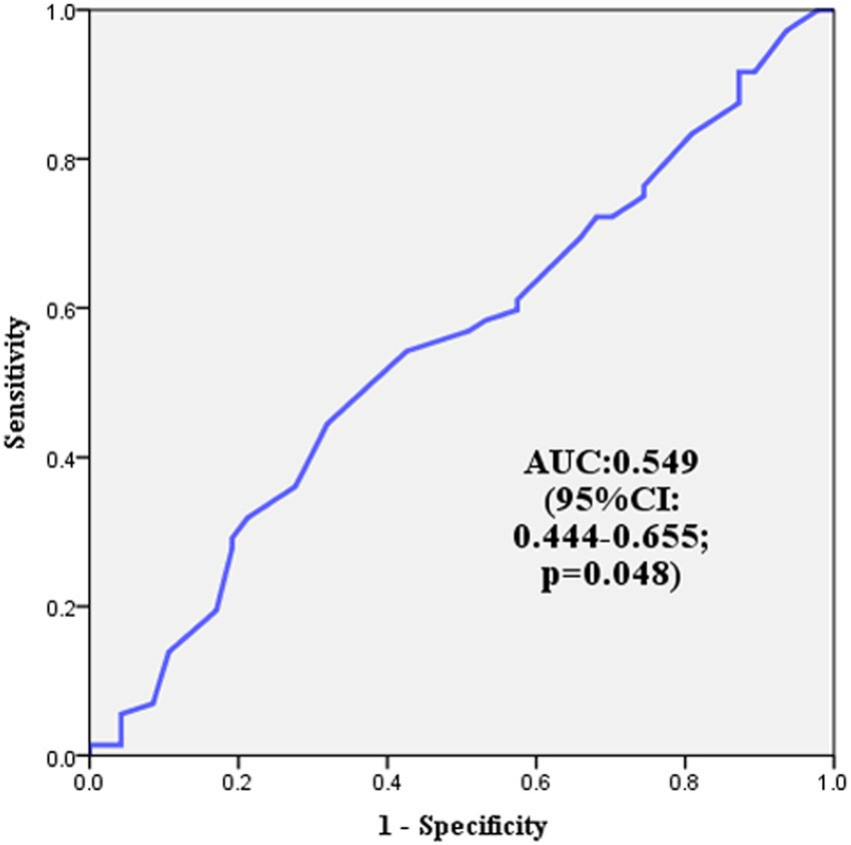
A representative Receiver operating characteristics (ROC) curve. The area under the curve (AUC) was used to calculate the slope of serum LDH status in the study.

In the second method (Method B), the serum LDH levels were measured at the surgery, before the first and fourth cycles of chemotherapy, and at the first follow-up after the chemotherapy. These time points corresponded to approximately at the surgery one month, three months, and seven months after the surgery, respectively. The values were used to calculate the slope of serum LDH changing curve by the least square method. Receiver operating characteristics (ROC) curves and the area under the curve (AUC) were used to determine the optimal cutoff points for the Method B. ROC curves, which help to choose the cut-points associated with optimal sensitivity and specificity, are commonly used in medical research to evaluate screening tests and identify thresholds to facilitate the decision making about patients^50^. Regarding the Method B in the TNBC patients, 5.0 U/L/month was identified as the optimal cutoff point for distinguishing the patients with a good prognosis from the patients with a poor prognosis (P = 0.048, AUC = 0.549), and the sensitivity and specificity were 44.4% and 68.1% (Fig. 3). All the patients were divided into two groups: the LDH high group (≥5.0 U/L/month) or the LDH low group (<5.0 U/L/month). In addition, formalin-fixed paraffin-embedded breast cancer tissues were obtained from the modified radical mastectomy before any systemic treatments.

### Statistical methods

All the data were analyzed with the statistical package SPSS (version 20.0 for Windows, Chicago, IL) software, and Pearson’s chi-square tests were applied to study the association between LDH-A/LDH and other clinicopathological features of the enrolled breast cancer patients. The least square method was used to calculate the slope of serum LDH status and ROC to give the best cut-off of Method B. The LDH-A expression between the cancer tissues and the matched normal tissues was analyzed by paired samples *t*-test. The Kappa test and McNemar test were used to examine the consistency of tumor LDH-A expression levels and the baseline serum LDH levels. Survival curves were generated with the Kaplan-Meier method, and Cox’s proportional hazards regression model was used to evaluate the hazards. Differences with p < 0.05 were considered statistically significant.

### Data Availability Statement

All data generated or analysed during this study are included in this published article.

## References

[1] Ishikawa M (2014). Simultaneous expression of cancer stem cell-like properties and cancer-associated fibroblast-like properties in a primary culture of breast cancer cells. Cancers.

[2] Long JP, Li XN, Zhang F (2016). Targeting metabolism in breast cancer: How far we can go?. World journal of clinical oncology.

[3] Sorlie T (2001). Gene expression patterns of breast carcinomas distinguish tumor subclasses with clinical implications. Proceedings of the National Academy of Sciences of the United States of America.

[4] Perou CM (2000). Molecular portraits of human breast tumours. Nature.

[5] Thompson PA (2011). Selective genomic copy number imbalances and probability of recurrence in early-stage breast cancer. PloS one.

[6] The Cancer Genome Atlas Network. Comprehensive molecular portraits of human breast tumours. *Nature* **490**, 61–70, doi:10.1038/nature11412 (2012). 10.1038/nature11412 PMC3465532 23000897

[7] Dong T (2016). Altered glycometabolism affects both clinical features and prognosis of triple-negative and neoadjuvant chemotherapy-treated breast cancer. Tumour biology: the journal of the International Society for Oncodevelopmental Biology and Medicine.

[8] Lin NU, Amiri-Kordestani L, Palmieri D, Liewehr DJ, Steeg PS (2013). CNS metastases in breast cancer: old challenge, new frontiers. Clinical cancer research: an official journal of the American Association for Cancer Research.

[9] Kennecke H (2010). Metastatic behavior of breast cancer subtypes. Journal of clinical oncology: official journal of the American Society of Clinical Oncology.

[10] Aversa C (2014). Metastatic breast cancer subtypes and central nervous system metastases. Breast (Edinburgh, Scotland).

[11] Kodack DP, Askoxylakis V, Ferraro GB, Fukumura D, Jain RK (2015). Emerging strategies for treating brain metastases from breast cancer. Cancer cell.

[12] Fokstuen T (2000). Radiation therapy in the management of brain metastases from breast cancer. Breast cancer research and treatment.

[13] Le Scodan R (2007). Brain metastases from breast carcinoma: validation of the radiation therapy oncology group recursive partitioning analysis classification and proposition of a new prognostic score. International journal of radiation oncology, biology, physics.

[14] Chen EI (2007). Adaptation of energy metabolism in breast cancer brain metastases. Cancer research.

[15] Hanahan D, Weinberg RA (2011). Hallmarks of cancer: the next generation. Cell.

[16] Choi J, Jung WH, Koo JS (2013). Metabolism-related proteins are differentially expressed according to the molecular subtype of invasive breast cancer defined by surrogate immunohistochemistry. Pathobiology: journal of immunopathology, molecular and cellular biology.

[17] Zhao YH (2009). Upregulation of lactate dehydrogenase A by ErbB2 through heat shock factor 1 promotes breast cancer cell glycolysis and growth. Oncogene.

[18] Pinheiro C (2011). GLUT1 and CAIX expression profiles in breast cancer correlate with adverse prognostic factors and MCT1 overexpression. Histology and histopathology.

[19] Yang Y (2014). Different effects of LDH-A inhibition by oxamate in non-small cell lung cancer cells. Oncotarget.

[20] Mirebeau-Prunier D (2013). Estrogen-related receptor alpha modulates lactate dehydrogenase activity in thyroid tumors. PloS one.

[21] Zhao D (2013). Lysine-5 acetylation negatively regulates lactate dehydrogenase A and is decreased in pancreatic cancer. Cancer cell.

[22] Shi M (2014). A novel KLF4/LDHA signaling pathway regulates aerobic glycolysis in and progression of pancreatic cancer. Clinical cancer research: an official journal of the American Association for Cancer Research.

[23] Cai Z (2010). A combined proteomics and metabolomics profiling of gastric cardia cancer reveals characteristic dysregulations in glucose metabolism. Molecular & cellular proteomics: MCP.

[24] Girgis H (2014). Lactate dehydrogenase A is a potential prognostic marker in clear cell renal cell carcinoma. Molecular cancer.

[25] Cui J (2014). FOXM1 promotes the warburg effect and pancreatic cancer progression via transactivation of LDHA expression. Clinical cancer research: an official journal of the American Association for Cancer Research.

[26] Yao F, Zhao T, Zhong C, Zhu J, Zhao H (2013). LDHA is necessary for the tumorigenicity of esophageal squamous cell carcinoma. Tumour biology: the journal of the International Society for Oncodevelopmental Biology and Medicine.

[27] Fantin VR, St-Pierre J, Leder P (2006). Attenuation of LDH-A expression uncovers a link between glycolysis, mitochondrial physiology, and tumor maintenance. Cancer cell.

[28] Serganova I (2011). Metabolic imaging: a link between lactate dehydrogenase A, lactate, and tumor phenotype. Clinical cancer research: an official journal of the American Association for Cancer Research.

[29] Motzer RJ (2013). Prognostic factors for survival in 1059 patients treated with sunitinib for metastatic renal cell carcinoma. British journal of cancer.

[30] Wan XB (2013). High pretreatment serum lactate dehydrogenase level correlates with disease relapse and predicts an inferior outcome in locally advanced nasopharyngeal carcinoma. European journal of cancer (Oxford, England: 1990).

[31] Mekenkamp LJ (2012). Mucinous adenocarcinomas: poor prognosis in metastatic colorectal cancer. European journal of cancer (Oxford, England: 1990).

[32] Giroux Leprieur E (2012). Factors associated with long-term survival of patients with advanced non-small cell lung cancer. Respirology (Carlton, Vic.).

[33] Lorch A (2010). Prognostic factors in patients with metastatic germ cell tumors who experienced treatment failure with cisplatin-based first-line chemotherapy. Journal of clinical oncology: official journal of the American Society of Clinical Oncology.

[34] Kamiya N (2014). Clinical outcomes by relative docetaxel dose and dose intensity as chemotherapy for Japanese patients with castration-resistant prostate cancer: a retrospective multi-institutional collaborative study. International journal of clinical oncology.

[35] He WZ (2013). Gamma-glutamyl transpeptidase level is a novel adverse prognostic indicator in human metastatic colorectal cancer. Colorectal disease: the official journal of the Association of Coloproctology of Great Britain and Ireland.

[36] Sau S, Biswas A, Roy A, Sau S, Ganguly S (2013). Retrospective analysis of the clinical and demographic variables on the outcomes after second-line treatment in advanced non-small cell lung cancer. Indian journal of medical and paediatric oncology: official journal of Indian Society of Medical & Paediatric Oncology.

[37] Kim HM, Jung WH, Koo JS (2014). Site-specific metabolic phenotypes in metastatic breast cancer. Journal of translational medicine.

[38] Colgan SM, Mukherjee S, Major P (2007). Hypoxia-induced lactate dehydrogenase expression and tumor angiogenesis. Clinical colorectal cancer.

[39] Koukourakis MI (2011). Prognostic and predictive role of lactate dehydrogenase 5 expression in colorectal cancer patients treated with PTK787/ZK 222584 (vatalanib) antiangiogenic therapy. Clinical cancer research: an official journal of the American Association for Cancer Research.

[40] Suh SY, Ahn HY (2007). Lactate dehydrogenase as a prognostic factor for survival time of terminally ill cancer patients: a preliminary study. European journal of cancer (Oxford, England: 1990).

[41] Park HJ, Lyons JC, Ohtsubo T, Song CW (1999). Acidic environment causes apoptosis by increasing caspase activity. British journal of cancer.

[42] Williams AC, Collard TJ, Paraskeva C (1999). An acidic environment leads to p53 dependent induction of apoptosis in human adenoma and carcinoma cell lines: implications for clonal selection during colorectal carcinogenesis. Oncogene.

[43] Augoff K, Grabowski K (2004). [Significance of lactate dehydrogenase measurements in diagnosis of malignancies]. Polski merkuriusz lekarski: organ Polskiego Towarzystwa Lekarskiego.

[44] Arora R (2015). Inhibition of the Warburg effect with a natural compound reveals a novel measurement for determining the metastatic potential of breast cancers. Oncotarget.

[45] Kolev Y, Uetake H, Takagi Y, Sugihara K (2008). Lactate dehydrogenase-5 (LDH-5) expression in human gastric cancer: association with hypoxia-inducible factor (HIF-1alpha) pathway, angiogenic factors production and poor prognosis. Annals of surgical oncology.

[46] Jovanovic S, Jovanovic A, Crawford RM (2007). M-LDH serves as a regulatory subunit of the cytosolic substrate-channelling complex *in vivo*. Journal of molecular biology.

[47] Porporato PE, Dhup S, Dadhich RK, Copetti T, Sonveaux P (2011). Anticancer targets in the glycolytic metabolism of tumors: a comprehensive review. Frontiers in pharmacology.

[48] Koukourakis MI, Giatromanolaki A, Sivridis E, Gatter KC, Harris AL (2006). Lactate dehydrogenase 5 expression in operable colorectal cancer: strong association with survival and activated vascular endothelial growth factor pathway–a report of the Tumour Angiogenesis Research Group. Journal of clinical oncology: official journal of the American Society of Clinical Oncology.

[49] O’Reilly KE (2006). mTOR inhibition induces upstream receptor tyrosine kinase signaling and activates Akt. Cancer research.

[50] Deyo RA, Centor RM (1986). Assessing the responsiveness of functional scales to clinical change: an analogy to diagnostic test performance. Journal of chronic diseases.

